# Enhancing erythritol production from crude glycerol in a wild-type *Yarrowia lipolytica* by metabolic engineering

**DOI:** 10.3389/fmicb.2022.1054243

**Published:** 2022-11-21

**Authors:** Shuling Yang, Xuewei Pan, Qiang Wang, Qinglan Lv, Xian Zhang, Rongzhen Zhang, Zhiming Rao

**Affiliations:** The Key Laboratory of Industrial Biotechnology, Ministry of Education, School of Biotechnology, Jiangnan University, Wuxi, Jiangsu, China

**Keywords:** *Yarrowia lipolytica*, erythritol, crude glycerol, metabolic engineering, glycerol kinase, transketolase, erythritol dehydrogenase

## Abstract

**Background:** Erythritol is a zero-calorie sweetener that is widely used in the food, pharmaceutical, and medical industries. Crude glycerol is the main by-product of biodiesel, and the effective utilization of crude glycerol will help to improve biodiesel viability. Previous studies on the production of erythritol from *Y. lipolytica* using crude glycerol as a carbon source have focused on optimizing the fermentation process of the mutant *Y. lipolytica* Wratislavia K1, while metabolic engineering has not been successfully applied.

**Results:** To this end, we engineered the yeast *Y. lipolytica* to increase the productivity of this strain. Wild strains tolerant to high concentrations of crude glycerol were screened and identified. A series of rational metabolic approaches were employed to improve erythritol production. Among them, the engineered strain Y-04, obtained by tandem overexpression of *GUT1* and *GUT2*, significantly increased glycerol assimilation by 33.3%, which was consistent with the results of RT-qPCR analysis. The effects of tandem overexpression of *GUT1*, *GUT2*, *TKL1,* and *TAL1* on erythritol synthesis were also evaluated. The best results were obtained using a mutant that overexpressed *GUT1*, *GUT2,* and *TKL1* and knocked out *EYD1*. The final Y-11 strain produced 150 g/l erythritol in a 5-L bioreactor with a yield and productivity of 0.62 g/g and 1.25 g/l/h, respectively. To the best of our knowledge, this is the highest erythritol yield and productivity from crude glycerol ever reported in *Y. lipolytica*.

**Conclusion:** This work demonstrated that overexpression of *GUT1*, *GUT2,* and *TKL1* and knockdown of *EYD1* could be used to improve crude glycerol utilization and erythritol synthesis in *Y. lipolytica*. The process parameters such as erythritol yield and productivity were significantly elevated, which is valuable for industrial applications. Crude glycerol, as a carbon source, could efficiently restrict the synthesis of by-products while enhancing the generation of erythritol, compared to glucose. This indicates considerable potential for synthesizing value-added products from crude glycerol by *Y. lipolytica*.

## Introduction

Erythritol is a sweet-tasting four-carbon sugar alcohol that occurs naturally in seaweed, fungi, fruit, and fermented food. Erythritol is not digested in humans, according to clinical trials and animal toxicological testing, and 90% of the ingested is excreted through renal processes (Munro et al., 1998; Qiu et al., 2020). Due to the unique nutritional properties of erythritol, erythritol is used as a functional sugar in food and is beneficial for diabetics and obesity. Erythritol is primarily obtained in industrial production using microbial synthesis methods. Currently, most strains used for industrial erythritol production are osmolytic yeasts, of which *Y. lipolytica* has a clear genetic background and proven tools for molecular modification (Ryu et al., 2000; Lee et al., 2002, 2010; Lin et al., 2010; Moon et al., 2010; Liu et al., 2015; Amaro and Nicaud, 2016). Most commonly, erythritol is produced from glucose and fructose by *Y. lipolytica*. Although some of these processes have been developed to an industrial scale, they have high costs for fermentation media and significant by-product concentrations, which make downstream processing more challenging (Saran et al., 2015; Cheng et al., 2018).

Most researchers have concentrated on improving erythritol production by screening for suitable mutants or optimizing the medium or culture conditions. Qiu et al. (2020) rapidly screened the mutants by a combination of UV and ARTP mutagenesis and achieved an erythritol concentration of 148 g/l. Rakicka et al. (2017) obtained the mutant *Y. lipolytica* MK1 by UV mutagenesis and investigated the optimal C: N ratio in the medium, which eventually enhanced the erythritol concentration to 113.1 g/l. Mirończuk et al. (2014) found that the mutant *Y. lipolytica* Wratislavia K1 obtained by acetate-negative mutation was suitable for producing large amounts of erythritol. In addition, a few records of *Y. lipolytica* producing erythritol from glycerol have been reported (Rakicka et al., 2016b; Carly et al., 2017; Mirończuk et al., 2019; Bilal et al., 2020; Mawire et al., 2020; Jagtap et al., 2021). Crude glycerol is the principal by-product of the manufacturing of biodiesel, an emerging alternative and sustainable fuel. With the rapid growth of biodiesel demand and production worldwide, the efficient utilization of crude glycerol is urgent. Developing sustainable processes to convert crude glycerol into value-added products is vital to reduce the cost of biodiesel. Recent studies have indicated that glycerol-induced high osmotic pressure can enhance erythritol production (Yang et al., 2014). Jagtap et al. (2021) revealed that using glycerol as a carbon source significantly increased erythritol yield. They also demonstrated that the use of glycerol was effective in reducing the by-product mannitol production. Moreover, employing glycerol rather than glucose results in higher erythritol yields, which increases the profitability of the production process (Rymowicz et al., 2008; Tomaszewska et al., 2012; Rywińska et al., 2013). However, current studies on erythritol production using crude glycerol as a carbon source have been devoted to optimizing the fermentation process of the mutant *Y. lipolytica* Wratislavia K1, and metabolic engineering has still not been successfully applied.

In this work, the strain *Y. lipolytica* Y01 capable of producing erythritol from crude glycerol was screened by UV mutagenesis. Then, rational metabolic engineering strategies were adopted to enhance erythritol production, including overexpression of *GUT1* and *GUT2* to improve glycerol assimilation, overexpression of *TKL1* and *TAL1* to increase the precursor pool, and knockdown of *EYD1* to prevent the catabolism of erythritol. This work lays the foundation for producing value-added products from crude glycerol through metabolic modification of wild-type *Y. lipolytica*.

## Materials and methods

### Strains and media

All strains used in this study are listed in Additional file 1: Supplementary Table S1. *Escherichia coli* were grown at 37°C with constant shaking in LB medium for plasmid propagation. The wild-strain *Y. lipolytica* 2,021,417 was used as the initial strain for modification. *Y. lipolytica* cells were cultured at 30°C in YPD medium for strain activation or SC medium for screening transformants. SC medium (g/L): yeast nitrogen base (YNB) 1.7, (NH_4_)_2_SO_4_ 5, glucose 20. Note: add appropriate antibiotics or nutrients (400 μg/ml hygromycin B or 700 μg/ml bleomycin or 0.1 g/l leucine) to the medium before inoculation or plate coating. High glycerol medium (g/L): crude glycerol 500, yeast extract 5. Medium glycerol medium (g/L): crude glycerol 300, yeast extract 5. Erythritol fermentation medium (g/L): crude glycerol 250, yeast extract 1, NH_4_Cl 5, KH_2_PO_4_ 0.25, MgSO_4_·7H_2_O 0.5. Erythritol fermentation medium (pure glycerol as a carbon source; g/L): pure glycerol 250, yeast extract 1, NH_4_Cl 5, KH_2_PO_4_ 0.25, MgSO_4_·7H_2_O 0.5. Erythritol fermentation medium (glucose as a carbon source; g/L): glucose 250, yeast extract 1, NH_4_Cl 5, KH_2_PO_4_ 0.25, MgSO_4_·7H_2_O 0.5. The crude glycerol in this work was obtained from biodiesel waste. The composition of crude glycerol is (v/v): glycerol 80–85%, sodium salt 2.0%, methanol 10–15%, other organic matter 2.5%, and water 2%. 0.1 mol/l lithium acetate: accurately weigh 1.02 g of lithium acetate, dissolve in 90 ml of distilled water, adjust pH 6.0 with acetic acid, then fix the volume to 100 ml, sterilize at 115°C for 20 min and store at −20°C. 40% PEG4000: accurately weigh 20 g of PEG4000, dissolve in 30 ml of 0.1 mol/l lithium acetate (pH 6.0), dissolve fully, fix the volume to 50 ml, sterilize at 115°C for 20 min and store at −20°C.

### Construction of plasmids

All plasmids and primers used in this study are listed in Additional file 1: Supplementary Tables S2, Figures S3, respectively. For the knockout of *Ku70* and *LEU2*, two primer pairs (*Ku70*-sg-1 and *LEU2*-sg-1) were designed to construct plasmids pCAS1yl-∆*Ku70* and pCAS1yl-∆*LEU2*, respectively. The *Ku70*-UP-F/R, *Ku70*-DOWN-F/R, *LEU2*-UP-F/R, and *LEU2*-DOWN-F/R primer pairs were used to amplify the upstream and downstream homologous arms of 1,000 bp each from *Y. lipolytica* 2,021,417 genomic DNA. The *hygB* gene from *Coccidioides posadasii* was synthesized in pUC-GW to construct pUC-GW-*hygB* by Genewiz (Suzhou, China) with codon optimization. The *hygB* gene cloned into *Kpn*I linearized pINA1269 using the *hygB*-F/R primer pair to generate plasmid pINA1269-*hygB*. The fragment of hp4d-*hygB*-XPR2t from pINA1269-*hygB* was then amplified using the hp4d-*hygB*-XPR2t-F/R primer pair. A fusion fragment *Ku70* UP-hp4d-*hygB*-XPR2t-*Ku70* DOWN and *LEU2* UP-hp4d-*hygB*-XPR2t- *LEU2* DOWN was subsequently generated. The fusion fragments were cloned into *Pme*I linearized pCAS1yl-∆*Ku70* and pCAS1yl-*∆LEU2* to generate pCAS2yl-∆*Ku70* and pCAS2yl-∆*LEU2*, respectively. The *LEU2* sgRNA expression cassette from pCAS2yl-∆*LEU2* was then amplified with *LEU2* F/R primer pair and subsequently cascaded with the *Ku70* sgRNA expression cassette in pCAS2yl-∆*Ku70* to obtain pCAS2yl-∆*Ku70*∆*LEU2* for *Y. lipolytica* Y01 transformation (Gao et al., 2016). *EYD1* was knocked out using bleomycin as a screening marker, and the plasmid construction was the same as the knockout of *Ku70* and *LEU2*.

For single gene overexpression, the construction of pINA1269-*GUT1* was used as an example. 1,512 bp DNA fragment of gene *GUT1* was PCR amplified using genomic DNA of strain *Y. lipolytica* 2021417 as a template, and primer pair *GUT1*-F/R, respectively. The purified *GUT1* fragment was then digested using *Kpn*I, and cloned at the corresponding sites of plasmid pINA1269 to generate pINA1269-*GUT1*. For tandem overexpression of two genes, the construction of pINA1269-*GUT1*-*GUT2* was used as an example. The hp4d-*GUT2*-XPR2t fragment was PCR amplified using pINA1269-*GUT2* as a template, and primer pair hp4d-*GUT2*-XPR2t-F/R, respectively. The *GUT1* fragment and hp4d-*GUT2*-XPR2t fragment were then fused to obtain the *GUT1*-hp4d-*GUT2*-XPR2t fragment. The purified *GUT1*-hp4d-*GUT2*-XPR2t fragment was digested using *Kpn*I, and cloned at the corresponding sites of plasmid pINA1269 to generate pINA1269-*GUT1*-*GUT2*. For tandem overexpression of three genes, the construction of pINA1269-*GUT1*-*GUT2*-*TKL1* was used as an example. The hp4d-*TKL1*-XPR2t fragment was PCR amplified using pINA1269-*TKL1* as a template, and primer pair hp4d-*TKL1*-XPR2t-F/R, respectively. The *GUT1*-hp4d-*GUT2*-XPR2t fragment and hp4d-*TKL1*-XPR2t fragment were then fused to obtain the *GUT1*-hp4d-*GUT2*-XPR2t-hp4d-*TKL1*-XPR2t fragment. The purified *GUT1*-hp4d-*GUT2*-XPR2t-hp4d-*TKL1*-XPR2t fragment was digested using *Kpn*I, and cloned at the corresponding sites of plasmid pINA1269 to generate pINA1269-*GUT1*-*GUT2-TKL1*. For the construction of pINA1269-*GUT1*-*GUT2*-*TKL1*-*TAL1*, the hp4d-*TAL1*-XPR2t fragment was PCR amplified using pINA1269-*TAL1* as a template, and primer pair hp4d-*TAL1*-XPR2t-F/R, respectively. The *GUT1*-hp4d-*GUT2*-XPR2t-hp4d-*TKL1*-XPR2t fragment and hp4d-*TAL1*-XPR2t fragment were then fused to obtain the *GUT1*-hp4d-*GUT2*-XPR2t-hp4d-*TKL1*-XPR2t-hp4d-*TAL1*-XPR2t fragment. The purified *GUT1*-hp4d-*GUT2*-XPR2t-hp4d-*TKL1*-XPR2t-hp4d-*TAL1*-XPR2t fragment was digested using *Kpn*I, and cloned at the corresponding sites of plasmid pINA1269 to generate pINA1269-*GUT1*-*GUT2-TKL1*-*TAL1*.

### Mutagenic library construction by UV mutagenesis

The wild-strain *Y. lipolytica* 2,021,417 was cultured in a YPD medium for 24 h, centrifuged at 9000 × g, and washed three times with PBS. Then, the cell suspension was exposed to UV light using Biosan UVC/T-M-AR (Biosan, Latvia) until the cell survival was less than 0.05%, and the cells were inoculated onto YPD plates at 30°C. After the mutagenic strains were cultured, the strains with larger and thicker colonies were selected, numbered, and inserted into a test tube for storage. In contrast, the corresponding strains were inoculated into 250 ml shake flasks (50 ml Erythritol fermentation medium) at 30°C, 220 r/min for 120 h, and the erythritol titers were determined.

### Yeast transformation

The lithium acetate method was used to transform *Y. lipolytica* as described previously (Gietz and Schiestl, 2007). In brief, a single colony of *Y. lipolytica* was inoculated into 10 ml of YPD and incubated with shaking at 30°C, 220 r/min for 8–10 h (OD_600_ ≈ 0.3 ~ 0.5). Cells were harvested by centrifugation at 6000 × g for 5 min. The cells were then washed with sterile water, resuspended the cells with 1 ml 0.1 mol/l lithium acetate and incubated for 10 min at room temperature for transformation. The transformation mix was added to the cells in the following order: 10 μl 10 mg/ml ssDNA, 1 μg linearized plasmid DNA, 700 μl 40% PEG4000. The centrifuge tube was vortexed until the cell pellet was completely mixed. Cells were incubated at 30°C for 30 min and then heat shocked in a water bath at 42°C for 30 min. Cells were centrifuged to remove the transformation mix and resuspended in 100 μl of sterile water. Cells were then plated on the appropriate selection agar plates. Colonies were verified by PCR and then selected for erythritol production.

### Shake flask and 5-L bioreactor fermentation

Seed culture was carried out in a 500 ml flask containing 50 ml of YPD medium on a rotary shaker at 30°C and 220 r/min for 20 h. An inoculum of 20% was introduced into a shake flask containing 30 ml of the Erythritol fermentation medium. Shake flasks were performed on a rotary shaker at 30°C and 220 r/min for 120 h. An inoculum of 20% was introduced into a bioreactor containing 1.6 l of the Erythritol fermentation medium. Batch cultivations were carried out in a 5-L fermenter (Baoxing Co., China) at 30°C with a working volume of 2 l. The aeration rate was fixed at 1.0 l/min. The stirrer speed was adjusted to 800 rpm and the dissolved oxygen concentration was maintained at 20–30%. pH was maintained automatically at 3.0 by the addition of 20% (w/v) of NaOH solution. All cultures were carried out in three replications.

### Determination of fermentation parameters

The concentrations of erythritol, mannitol, arabinitol, and glycerol were determined by HPLC (Agilent 1,200 series; Agilent Technologies). The sample volume was 20 μl using an amino column 70 Å NH_2_ (250 × 4.6 mm; 5 μm) eluted with 80% acetonitrile as the mobile phase at a flow rate of 1.0 ml/min. The detector was a RID detector coupled to a detector temperature of 35°C and a column temperature of 40°C. The mass yield (Y_ERY_) and volumetric productivity (Q_ERY_) of erythritol and glycerol consumption were calculated based on previous studies (Mawire et al., 2020; Wang et al., 2020).

### RNA isolation and quantitative PCR analysis

Total RNA was extracted from *Y. lipolytica* according to the manufacturer’s instructions using the Bead-Beat Total RNA Mini kit (A&A Biotechnology, Gdynia, Poland). The TranScriba kit (A&A Biotechnology, Gdynia, Poland) was used to synthesize the cDNA strands.

Quantitative PCR (qPCR) was performed in a 7,500 real-time PCR thermal cycler (Applied Biosystems, Waltham, MA, USA) using an SYBR®Green B PCR MasterMix (A&A Biotechnology, Gdynia, Poland). Each reaction contained 0.5 μl of cDNA template, 5 μl of SYBR®Green B PCR MasterMix, and 0.5 μl of forwarding and reversed primers, which were made up to 10 μl using ddH_2_O. Reaction conditions were as follows:95°C for 3 min, 95°C for 15 s, 60°C for 30 s and 72°C for 30 s, 2–4 steps of 40 cycles, and melting curve phases: 94°C for 15 s, 60°C for 60 s, 95°C for 30 s, 60°C for 15 s. Each qPCR reaction was performed in technical replicates.

## Results

### Achievement of erythritol-producing strain by UV mutagenesis

**Figure 1:**
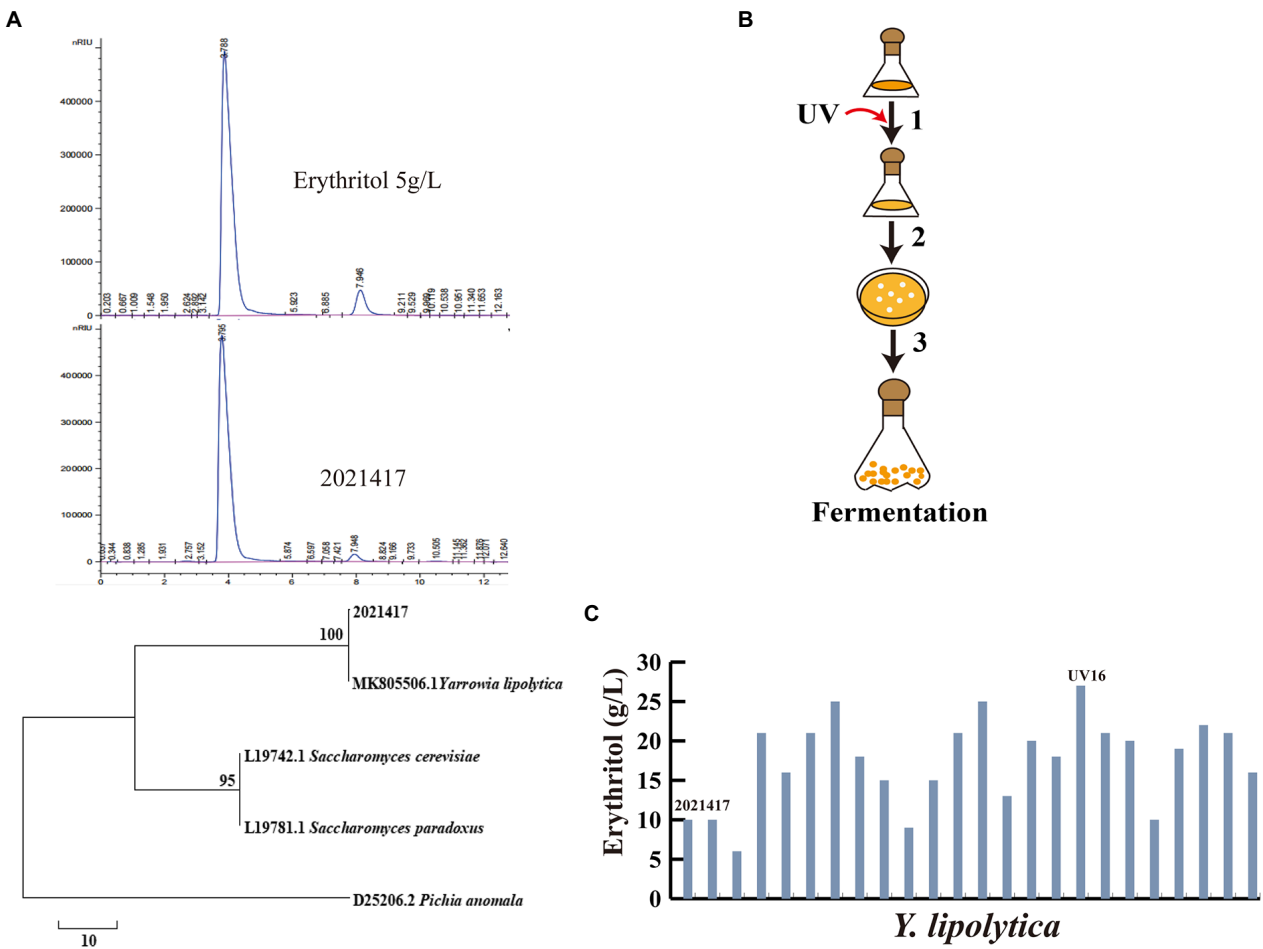
Identification and screening of mutant strains for erythritol production. **(A)** HPLC and phylogenetic tree of 2,021,417. **(B)** UV mutagenesis and mutant screening flowchart. 1: UV mutagenesis; 2: Growth of surviving cells on YPD plates; 3: Fermentation of strains obtained by mutagenesis. **(C)** Erythritol titers after UV mutagenesis. Three biological replicates were used to obtain the data. The error bars represented the standard deviation.

According to recent studies, highly osmotolerant yeast strains isolated from the daily activities of honey bees were the major source of erythritol-producing strains (Moon et al., 2010). To obtain effective strains for erythritol production, we screened surviving strains from honey with high glycerol medium and inoculated them into medium glycerol medium for fermentation. The erythritol-producing strain 2,021,417 was screened and analyzed by HPLC. It was sequenced and identified as *Y. lipolytica*, producing 10 ± 0.07 g/l erythritol in shake flask fermentation (Figure 1A). To further enhance the erythritol production capacity of *Y. lipolytica* 2,021,417, UV mutagenesis was first performed (Figure 1B). The strains obtained by UV mutagenesis were fermented in a shake flask. The fermentation results are shown in Figure 1C. The erythritol production of some strains decreased after UV mutageneses, such as UV-2 and UV-9. Meanwhile, the erythritol production was also increased in most strains, such as UV-3, UV-5, UV-6, UV-11, UV-12, UV-16, UV-17, UV-21, and UV-22 reached more than 20 g/l, among which UV-16 had the highest erythritol titer of 27 ± 0.03 g/l. Compared to the initial strain 2,021,417, the erythritol titer of UV-16 increased by 170%. Therefore, UV-16 was renamed *Y. lipolytica* Y01 for further metabolic engineering.

### Construction of a chassis strain derived from mutant *Yarrowia lipolytica* Y01

**Figure 2:**
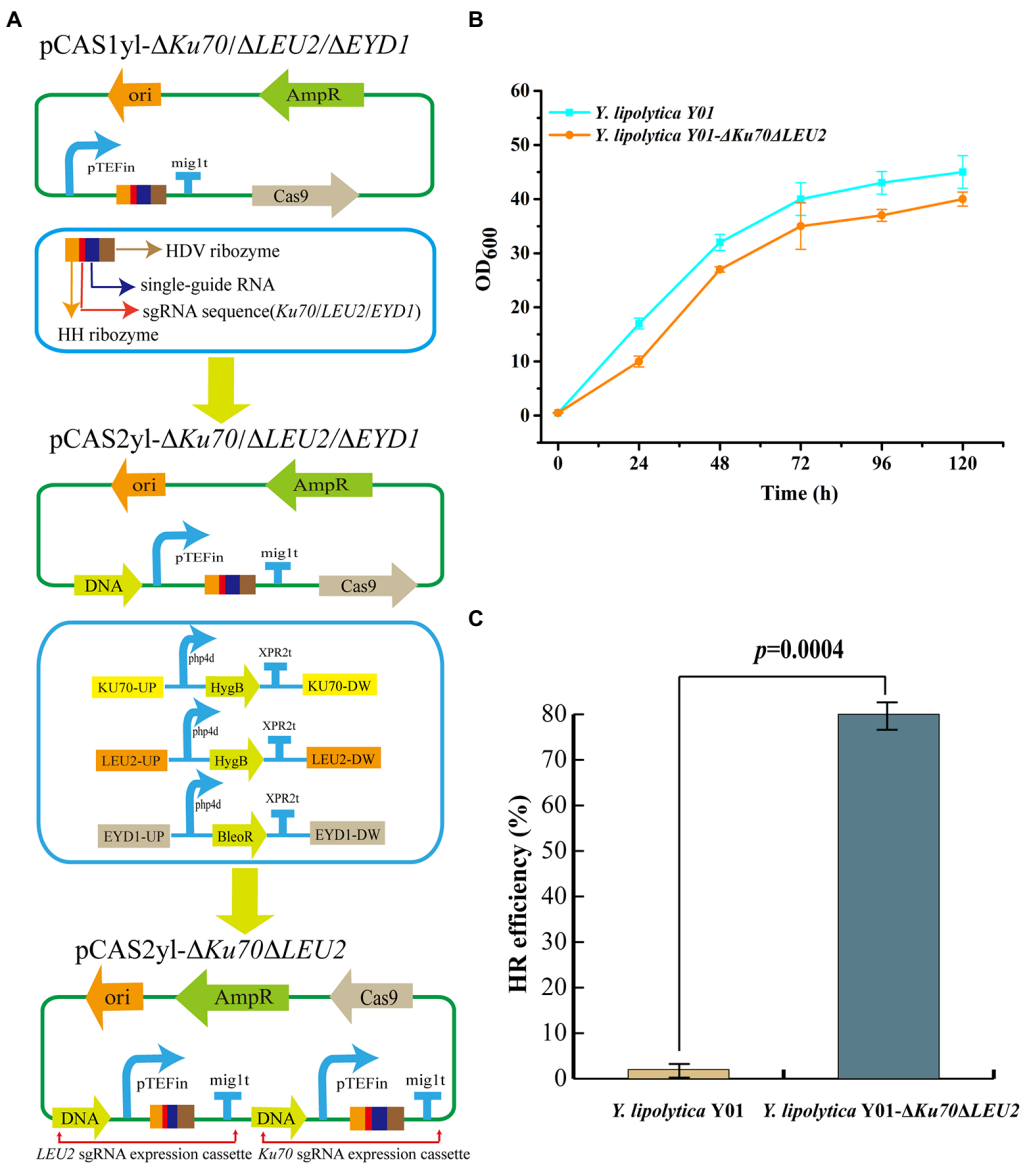
Influence of *Ku70* and *LEU2* knockout on strain growth and HR efficiency. **(A)** Construction of *Ku70* and *LEU2* knockout plasmids. pTEFin: Strong endogenous promoter; mig1t: Terminator; HDV ribozyme, single-guide RNA, single sequence and HH ribozyme were used to construct sgRNA expression cassette; DNA: Donor DNA. **(B)** Growth curves of *Yarrowia lipolytica* Y01 and *Y. lipolytica* Y01-Δ*Ku70*Δ*LEU2* strains. **(C)** Efficiencies of gene integration in *Y. lipolytica* Y01 and *Y. lipolytica* Y01-Δ*Ku70*Δ*LEU2* strains. Three biological replicates were used to obtain the data. The error bars represented the standard deviation. Significance (*p*-value) was evaluated by two-sided *t*-test.

A mutant *Y. lipolytica* Y01 was obtained by UV mutagenesis. Next, metabolic engineering will be combined to improve erythritol production further. First, the antibiotic markers were screened for *Y. lipolytica* Y01 knockout to create a chassis strain that could be genetically modified (The screening of antibiotics is listed in Additional file 1: Supplementary Figure S1). Non-homologous end joining (NHEJ) and homologous recombination (HR) are the main genome engineering approaches used by *Y. lipolytica*. Due to non-specific NHEJ, HR in *Y. lipolytica* is limited in terms of integration efficiency and length (Abdel-Mawgoud and Stephanopoulos, 2020). The HR efficiency of short-length flanking fragments was enhanced when the *Ku70* gene responsible for repairing DNA double-strand breaks (DSBs) in the NHEJ pathway was absent (Verbeke et al., 2013). Therefore, the gene *Ku70* was knocked out to improve integration efficiency. Furthermore, since the integration plasmid used in this work was leucine back-complemented, the auxotrophic strain for leucine was constructed by knocking out gene *LEU2*. Hygromycin B was used as a screening marker to knockout genes *Ku70* and *LEU2*, laying the foundation for later molecular manipulation (Figure 2A). The *Y. lipolytica* Y01-Δ*Ku70*Δ*LEU2* and *Y. lipolytica* Y01 were incubated in Erythritol fermentation medium for 120 h, and their growth curve was determined. The results showed that the growth trends of *Y. lipolytica* Y01-Δ*Ku70*Δ*LEU2* and *Y. lipolytica* Y01 were similar, which indicated that the knockout of *Ku70* and *LEU2* had no negative effects on fitness (Figure 2B). In addition, the conversion rate of positive transformants was 2% (1/50) when the 7.2 kb DNA fragment was integrated into the *Y. lipolytica* Y01 genome, whereas the conversion rate reached 80% (40/50) for *Y. lipolytica* Y01-Δ*Ku70*Δ*LEU2* (Figure 2C). Next, we will construct and optimize the erythritol-producing strains based on *Y. lipolytica* Y01-Δ*Ku70*Δ*LEU2*.

### Improve glycerol assimilation in the strain *Yarrowia lipolytica* Y01-Δ*Ku70*Δ*LEU2* and erythritol production

**Figure 3:**
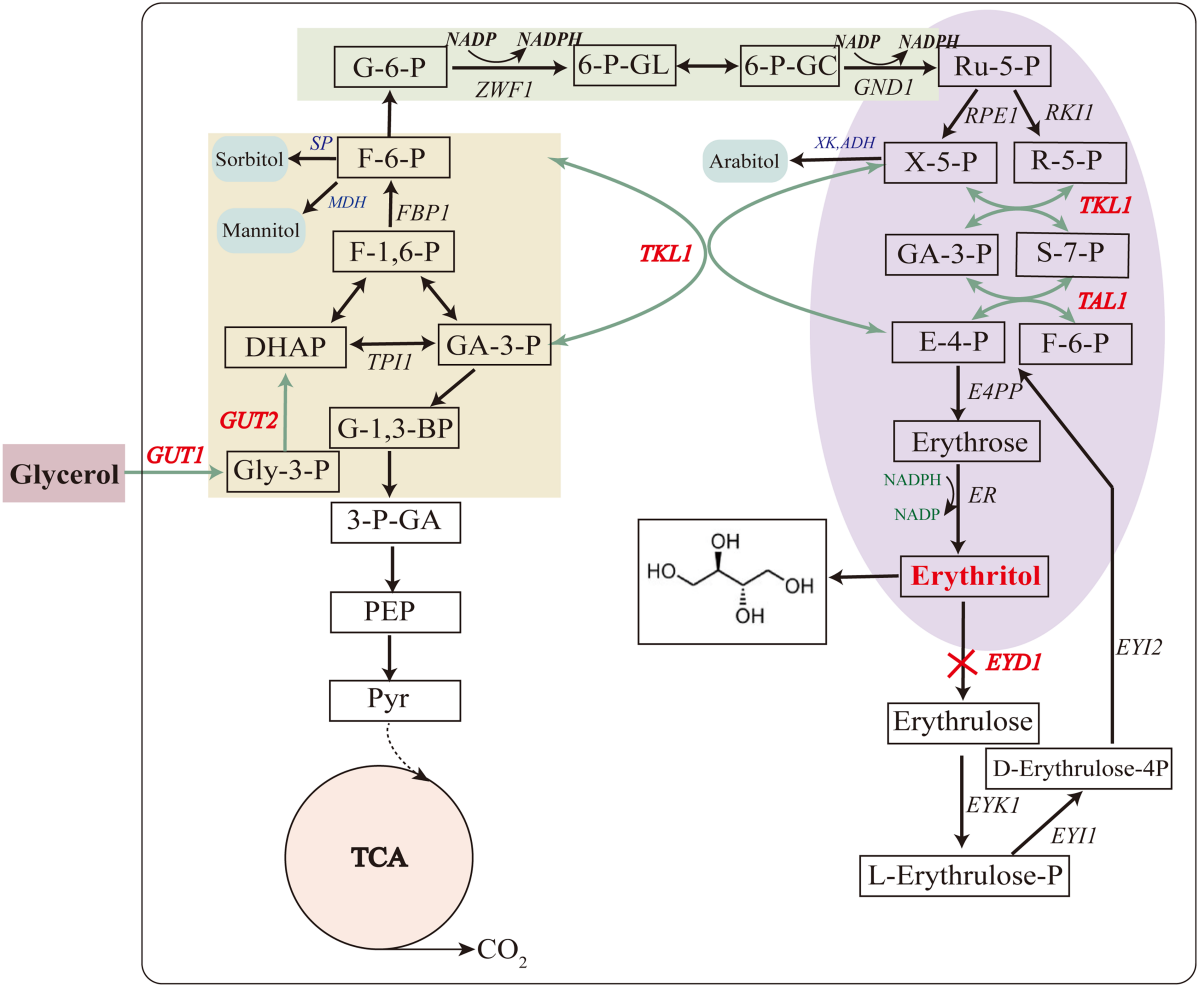
Metabolic pathway of erythritol biosynthesis on glycerol in *Y. lipolytica*. Genes marked in red and bold were selected for overexpression (except for *EYD1*) or disruption (*EYD1*). *GUT1* glycerol kinase, *GUT2* glycerol 3-phosphate dehydrogenase, *TKL1* transketolase, *TAL1* transaldolase, *EYD1* erythritol dehydrogenase.

**Figure 4:**
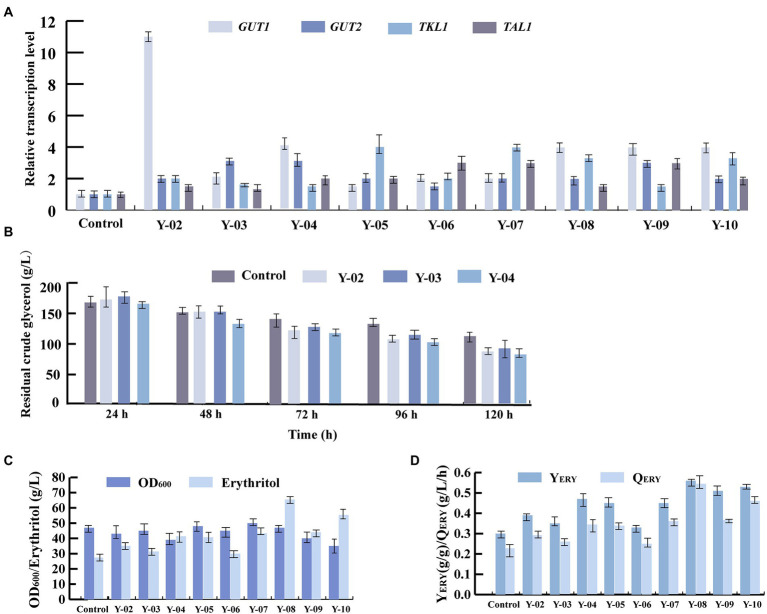
Four key genes (*GUT1*, *GUT2*, *TKL1*, *TAL1*) involved in the synthesis of the erythritol pathway from glycerol were overexpressed individually or in tandem in *Y. lipolytica* Y01-Δ*Ku70*Δ*LEU2*. **(A)** Relative transcription level of *GUT1*, *GUT2*, *TKL1,* and *TAL1* in different engineered strains. **(B)** Crude glycerol assimilation in different engineered strains. **(C)** Erythritol titers and OD_600_ in different engineered strains. **(D)** Erythritol yield and productivity in different engineered strains. Three biological replicates were used to obtain the data. The error bars represented the standard deviation.

Glycerol kinase and glycerol-3-P dehydrogenase are encoded by *GUT1* (YALI0F00484g) and *GUT2* (YALI0B13970g), respectively. These two enzymes are mainly involved in glycerol assimilation in the erythritol synthesis pathway (Figure 3; Mirończuk et al., 2015). To improve the glycerol assimilation of *Y. lipolytica* Y01-Δ*Ku70*Δ*LEU2*, we obtained strains Y-02, Y-03 and Y-04 by overexpressing *GUT1* and *GUT2* separately and in tandem. We first evaluated the expression levels of *GUT1* and *GUT2* by RT-qPCR of total RNA. 18sRNA was used as the reference gene. According to the analysis, the Y-02, Y-03, and Y-04 strains had higher expression levels of *GUT1* and *GUT2* (Figure 4A). Notably, both *GUT1* and *GUT2* expression levels were raised by overexpressing either *GUT1* or *GUT2*, with an 11-fold increase in *GUT1* expression in strain Y-02 (pINA1269-*GUT1*). However, the expression levels of *GUT1* and *GUT2* were only slightly up-regulated in the co-expression strain, which may be due to the mutual coordination between genes to maintain the dynamic balance of the cells. The results were similar to those obtained in previous studies (Mirończuk et al., 2016; Martău et al., 2020). Based on these results, the erythritol titer, Y_ERY_, Q_ERY_, and glycerol assimilation of the engineered strains were determined by shaking the flask. The results of the shake flask experiments are summarized in Figures 4B–D. The results indicated that there was no significant different in crude glycerol consumption before 48 h. After 48 h, the crude glycerol consumption of all engineered strains increased rapidly. In strain Y-04 (pINA1269-*GUT1*-*GUT2*), crude glycerol consumption was increased by 33% compared to the control strain (*Y. lipolytica* Y01-Δ*Ku70*Δ*LEU2*; Figure 4B). Mirończuk et al. (2016) also found that glycerol consumption was higher in *Y. lipolytica* strain Y101 with tandem overexpression of *GUT1* and *GUT2*. Carly et al. (2017) overexpressed *GUT1* and *GUT2* separately or in tandem for *Y. lipolytica* strain Po1d and found that the engineered strain overexpressing *GUT1* had a higher specific glycerol consumption rate. This may be due to the different expression levels of the same gene in different hosts. Furthermore, we found that crude glycerol consumption of the engineered strains increased rapidly after 48 h. As the synthesis of erythritol was regulated by growth, the cells were induced to produce erythritol after the culture reached a stationary phase at about 48 h. The rapid increase in crude glycerol consumption indicated that crude glycerol consumption and erythritol synthesis were interrelated.

For strains Y-02 (pINA1269-*GUT1*) and Y-03(pINA1269-*GUT2*), the erythritol titers were 35 g/l and 31 g/l, which increased by 29.6 and 14.8% compared to the control strain, achieving Y_ERY_ 0.39 g/g and 0.35 g/g, Q_ERY_ 0.292 g/l/h and 0.258 g/l/h, respectively (Figures 4C,D). Previous research discovered that erythritol synthesis and the conversion of erythritol to glycerol were significantly affected by the overexpression of *GUT1*. Still, overexpression of *GUT2* only affected glycerol consumption of the engineered strain and did not significantly improve erythritol synthesis, which was verified by our results (Mirończuk et al., 2016; Carly et al., 2017). The highest erythritol titer, Y_ERY_, and Q_ERY_ were achieved in strain Y-04 (pINA1269-*GUT1*-*GUT2*). For strain Y-04, the erythritol titer, Y_ERY,_ and Q_ERY_ were 51.9, 56.7, and 52% higher than the control strain (41 and 27 g/l, 0.47 and 0.30 g/g, 0.342 and 0.225 g/l/h, respectively; Figures 4C,D). A significant increase in all process parameters could be found for strain Y-04, which further demonstrated that the tandem overexpression of *GUT1* and *GUT2* not only enhanced crude glycerol consumption but also improved erythritol synthesis.

### Improve precursor supply in the strain *Yarrowia lipolytica* Y01-Δ*Ku70*Δ*LEU2* and erythritol production

Transketolase and transaldolase, which are primarily involved in the supply of precursors in the erythritol synthesis pathway, are encoded by the genes *TKL1* (YALI0E06479g) and *TAL1* (YALI0F15587g), respectively (Figure 3; Mirończuk et al., 2017). Thus, to further enhance precursors supply, *TKL1* and *TAL1* were overexpressed in *Y. lipolytica* Y01-Δ*Ku70*Δ*LEU2*, and engineered strains Y-05, Y-06, and Y-07 were obtained. The RT-qPCR results of total RNA showed that the relative expression levels of *GUT1*, *GUT2*, *TKL1,* and *TAL1* were increased in all engineered strains, which was consistent with the overexpression results of *GUT1* and *GUT2* (Figure 4A).

Based on these results, we evaluated the effect of *TKL1* and *TAL1* overexpression on erythritol production. For strain Y-05 (pINA1269-*TKL1*), the erythritol titer, Y_ERY_ and Q_ERY_ increased to 40 g/l, 0.45 g/g and 0.333 g/l/h, which were increased by 48.1, 50 and 48% compared to the control strain, respectively (Figure 4C, D). This suggested that transketolase (*TKL1*) is more important in promoting erythritol synthesis. Carly et al. (2017) found that overexpression of *TKL1* obtained the best results in terms of reduced fermentation time and improved erythritol production compared to overexpression of other genes. In addition, Mirończuk et al. (2017) discovered that transketolase was a key enzyme for erythritol synthesis in *Y. lipolytica*, and overexpression of *TKL1* resulted in a 2-fold improvement in erythritol synthesis. In light of these findings, we further investigated the effect of tandem overexpression of multiple genes on erythritol synthesis.

### The pull and push strategy to enhance erythritol production

As mentioned above, tandem overexpression of *GUT1* and *GUT2* enhanced crude glycerol assimilation and improved erythritol synthesis in the engineered strain. Overexpression of *TKL1* significantly increased erythritol titer, Y_ERY_, and Q_ERY_. Co-expression of multiple key genes has been shown to improve cellular metabolic performance. Therefore, to further enhance erythritol production, *GUT1*, *GUT2,* and *TKL1* or *GUT1*, *GUT2,* and *TAL1* were overexpressed in tandem in strains Y-08 and Y-09. For strain Y-08, the erythritol titer, Y_ERY_, and Q_ERY_ reached 65 g/l, 0.56 g/g, and 0.541 g/l/h, which were increased by 58.5, 19.2, and 58.2% compared to the control strain Y-04, respectively (Figures 4C,D). This suggested that the primary rate-limiting processes in erythritol synthesis were glycerol assimilation and precursor supply, and enhancing these reactions might assist increase erythritol production. To further develop this push and pull strategy, *GUT1*, *GUT2*, *TKL1,* and *TAL1* were overexpressed in tandem to obtain the engineered strain Y-10. For strain Y-10 (pINA1269-*GUT1*-*GUT2*-*TKL1*-*TAL1*), erythritol titer, Y_ERY_, and Q_ERY_ were reduced by 15.3, 9.1, and 15.3% compared to the strain Y-08 (pINA1269-*GUT1*-*GUT2*-*TKL1*; Figures 4C,D). Overexpression of *TAL1* with *GUT1*, *GUT2* and *TKL1* in tandem could not further enhance erythritol production. Therefore, overexpression of *GUT1*, *GUT2,* and *TKL1* in tandem was the combination that maintained the highest erythritol titer, Y_ERY_, and Q_ERY_.

### Disruption of *EYD1* in strain Y-08 further increases erythritol production

**Figure 5:**
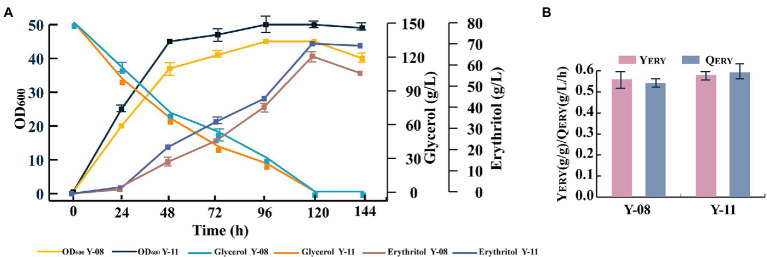
The *EYD1* gene encoding erythritol dehydrogenase was disrupted in strain Y-11. **(A)** OD_600_, crude glycerol utilization and erythritol titers of engineered strains Y-08 and Y-11. **(B)** Erythritol yield and productivity of engineered strains Y-08 and Y-11. Three biological replicates were used to obtain the data. The error bars represented the standard deviation.

Although *Y. lipolytica* can produce high levels of erythritol, it is also capable of consuming erythritol as a carbon source. This ability has a negative impact on erythritol productivity and is a serious drawback for the development of high erythritol-producing strains. The *EYD1* (YALI0F01650g) gene encoding erythritol dehydrogenase is involved in the catabolic pathway of erythritol (Carly et al., 2018). To prevent the catabolism of erythritol, the *EYD1* gene was knocked out and strain Y-11 was obtained. As expected, strain Y-11 was unable to use erythritol in a crude glycerol-deficient medium, whereas strain Y-08 was able to begin consuming erythritol after crude glycerol depletion (Figure 5A). As a result, it can be inferred that *EYD1* is involved in the catabolic pathway of erythritol, and the knockdown of *EYD1* effectively inhibited the catabolism of erythritol. In addition, strain Y-11 had better erythritol production performance. Compared to strain Y-08, the erythritol titer, yield, and productivity were increased by 9.4, 3.6, and 9.2%, respectively (Figure 5B).

### Batch bioreactor fermentation

**Figure 6:**
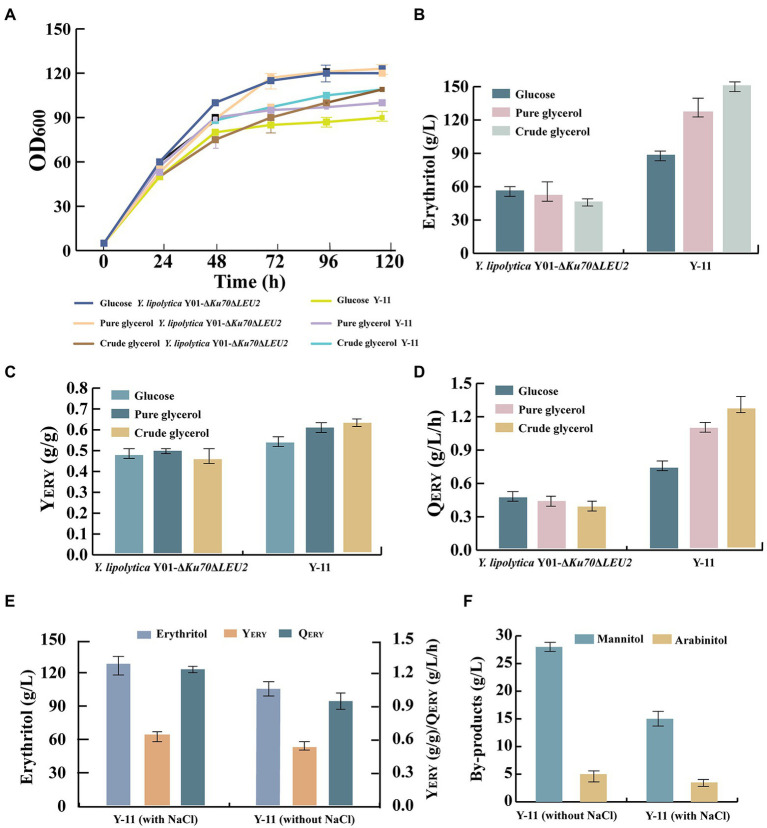
The parameters of the processes conducted in batch bioreactor cultivation. **(A)** OD_600_ of strains *Y. lipolytica* Y01-Δ*Ku70*Δ*LEU2* and Y-11 in different carbon sources. **(B)** Erythritol titers of strains *Y. lipolytica* Y01-Δ*Ku70*Δ*LEU2* and Y-11 in different carbon sources. **(C)** Erythritol yield of strains *Y. lipolytica* Y01-Δ*Ku70*Δ*LEU2* and Y-11 in different carbon sources. **(D)** Erythritol productivity of strains *Y. lipolytica* Y01-Δ*Ku70*Δ*LEU2* and Y-11 in different carbon sources. **(E)** Erythritol titers, yield and productivity of Y-11with NaCl or without NaCl. **(F)** The main by-products of Y-11 with NaCl or without NaCl. Three biological replicates were used to obtain the data. The error bars represented the standard deviation.

Previous experiments have shown that engineered strain Y-11 was the most capable of producing erythritol from crude glycerol. Next, we investigated whether the Y-11 producer capabilities could be fully utilized for large-scale production. In the 5-L bioreactor, the capacity of Y-11 and control strain (*Y. lipolytica* Y01-Δ*Ku70*Δ*LEU2*) to produce erythritol was evaluated. Cell growth, crude glycerol consumption, erythritol titer, Y_ERY_, and Q_ERY_ of the fermentation process were monitored (Figure 6). There was no significant difference in cell growth between engineered strain Y-11 and the control strain. For strain Y-11, it rapidly grew to a maximum OD_600_ of 109 and reached a stable phase at 48 h. The crude glycerol consumption of strain Y-11 was similar to that of the control strain before 48 h. After 48 h, the crude glycerol consumption of Y-11 increased rapidly, which was consistent with the results of the shake flask experiments. The erythritol titer, Y_ERY_, and Q_ERY_ of Y-11 reached 150 g/l, 0.62 g/g, and 1.25 g/l/h, which increased by 172.7, 31.9, and 179.4% compared to the control strain, respectively, indicating that appropriate metabolic modification greatly improved crude glycerol consumption, erythritol titer, Y_ERY_ and Q_ERY_ of *Y. lipolytica* (Figure 6). In addition, the results demonstrated that the erythritol titer, Y_ERY_, and Q_ERY_ in the 5-L bioreactor were increased by 111, 7, and 111% compared to that obtained in shake flasks, respectively. It was identified that erythritol could be produced more efficiently in the 5-L bioreactor for large-scale production, which may be due to the high dissolved oxygen requirement of *Y. lipolytica* during the fermentation process. The limited dissolved oxygen in the shake flasks cannot meet the needs during the fermentation process.

Crude glycerol, a mixture of glycerol and other substances, was produced as a by-product along with biodiesel production. It is unclear if crude glycerol contains components that inhibit the growth and product production of engineered strains. To determine whether crude glycerol affects the erythritol production by the engineered strain Y-11, we fermented Y-11 and control strain under different carbon sources (Figure 6). The results revealed no significant difference in the growth of Y-11 and control strain in glucose, pure glycerol, and crude glycerol. In contrast, the erythritol titer, Y_ERY_, and Q_ERY_ of Y-11 in crude glycerol were slightly increased compared to the glucose and pure glycerol. These results indicated that the waste substrate in crude glycerol had no negative effect on the cell growth and erythritol synthesis of strain Y-11. Furthermore, Y-11 was fermented with and without NaCl. The results revealed that the addition of NaCl effectively reduced the by-products mannitol and arabinitol by 46 and 30%, mainly because increased osmotic pressure affected the proportions of erythritol, mannitol, and arabinitol produced by *Y. lipolytica*. The same results were also obtained in previous studies (Mirończuk et al., 2017; Bilal et al., 2020).

## Discussion

There have been many studies using classical mutagenesis methods to isolate new microbial strains for obtaining high yields of target products. The mutant *Y. lipolytica* MK1 obtained by ultraviolet mutation showed excellent performance in erythritol production (Mirończuk et al., 2015). Many mutants with enhanced erythritol production were obtained by chemical mutagenesis of *Moniliella* sp. 440 (Lin et al., 2010). This work used UV mutation to generate a strain of *Y. lipolytica* that can produce erythritol from crude glycerol, and its erythritol output was 170% higher than that of the wild strain.

*GUT1* and *GUT2* genes were overexpressed singly and in tandem to improve crude glycerol utilization. Surprisingly, the crude glycerol consumption of all engineered strains increased rapidly after 48 h and was superior to the control strain. This result indicated that crude glycerol utilization and erythritol synthesis were coordinated with each other. Overexpression of *GUT1* and *GUT2* improved crude glycerol utilization and shortened the fermentation time of the product, which can effectively reduce industrial costs. This has been discovered in previous studies on the metabolic modifications of *Y. lipolytica* (Tai and Stephanopoulos, 2013). Shake flask experiments were performed on engineered strains to understand the coordination between rapid glycerol utilization and enhanced metabolites. The expression level of *GUT1* was significantly increased, and erythritol synthesis was also improved when the *GUT1* gene was overexpressed. A similar result was observed previously (Mirończuk et al., 2016). However, we found that overexpression of the *GUT2* gene did not improve erythritol synthesis. Mirończuk et al. (2016) found that overexpression of the *GUT2* gene resulted in half erythritol production and increased citric acid production, possibly because overexpression of *GUT2* redefined carbon flow to the TCA cycle. It has also been demonstrated that the knockdown of *GUT2* increased fatty acid synthesis in *Y. lipolytica* (Dulermo and Nicaud, 2011). The engineered strain overexpressing *GUT1* and *GUT2* exhibited excellent erythritol production capacity. The erythritol titer, Y_ERY_, and Q_ERY_ increased by 51.9, 56.7, and 52% compared to the control strain, respectively, but the biomass decreased slightly. The results described above might be explained by the fact that overexpression of *GUT1* requires increased ATP levels, and that overexpression of *GUT2* results in an excess of NADH that is utilized for ATP production by glycerol kinase *via* oxidative phosphorylation. The synthesis of large amounts of ATP inhibits the TCA cycle, thus allowing carbon flow into the pentose phosphate pathway and enhancing erythritol synthesis (Wang et al., 2015).

We explored different metabolic engineering strategies to improve erythritol production. This work focused on improving the utilization of crude glycerol and the supply of precursors. It was found that single gene overexpression of *GUT1*, *GUT2*, *TKL1,* and *TAL1* increased erythritol production. When the *TKL1* gene was overexpressed, the erythritol titer, Y_ERY_, and Q_ERY_ of the engineered strain were significantly increased by 48.1, 50, and 48% compared to the control strain. This suggested that the *TKL1* gene encoding transketolase plays a crucial role in erythritol synthesis. Overexpression of *TKL1* in *Y. lipolytica* Po1d strain increased erythritol titer by 16% compared to the wild strain, and yield increased to 0.59 g/g (Carly et al., 2017). Mirończuk et al. (2017) functionally overexpressed *TKL1*, *TAL1*, *ZWF1,* and *GND1*, among which *TKL1* overexpression increased erythritol production by 2 times. However, Jagtap et al. (2021) did not find that overexpression of the *TKL1* gene in the *Y. lipolytica* PO1f strain increased erythritol, possibly because the *Y. lipolytica* PO1f strain is an uracil and leucine auxotrophic strain, which somehow limits erythritol production.

We also evaluated the effect of tandem overexpression of *GUT1*, *GUT2*, *TKL1,* and *TAL1* on erythritol synthesis. The results showed that the maximum erythritol titer, Y_ERY_, and Q_ERY_ were obtained when *GUT1*, *GUT2*, and *TKL1* were overexpressed in tandem. To demonstrate the practicability of the engineered strain, it was fermented in the 5-L bioreactor for 120 h. Table 1 lists the main parameters of erythritol production in previous studies and this work. Erythritol is currently produced commercially entirely by fermentation of substrates containing sugars, such as glucose and fructose. Although fermentation is effective, the expensive substrate and high concentration of by-products limit the large-scale production of erythritol (Ghezelbash et al., 2014; Cheng et al., 2018; Wang et al., 2020). Applying alternate substrates is a typical strategy to further reduce costs. There are many studies on glycerol as a new carbon source for erythritol fermentation, including pure and crude glycerol. Currently, most studies focus on the fermentation of pure glycerol by the *Y. lipolytica* strain to produce erythritol (Mirończuk et al., 2014; Tomaszewska et al., 2014; Yang et al., 2014; Rywińska et al., 2015; Rakicka et al., 2016a; 2017 Carly et al., 2017; Janek et al., 2017; Mirończuk et al., 2017;). Previous studies on erythritol production using crude glycerol as a carbon source have focused on optimizing the fermentation process of the mutant *Y. lipolytica* Wratislavia K1 (Rymowicz et al., 2008; Tomaszewska et al., 2012; Mirończuk et al., 2014; Rakicka et al., 2016b; Jagtap et al., 2021). Jagtap et al. (2021) first used metabolic engineering to overexpress the *PYP*, *GUT1*, and *TKL1* genes to achieve an erythritol titer of 16.7 g/l in shake flask experiments. However, no significant erythritol production was observed during batch culture in a bioreactor. This was most likely caused by unknown contaminants in crude glycerol, which prevented the synthesis of the product. In this work, the engineered strain *Y. lipolytica* Y-11 was obtained by overexpressing *GUT1*, *GUT2* and *TKL1* genes and knocking out the *EYD1* gene. To determine whether crude glycerol affects the erythritol production of the engineered strain Y-11, we fermented Y-11 under different carbon sources. The results showed a slight increase in erythritol titer, Y_ERY_ and Q_ERY_ of Y-11 in crude glycerol compared to glucose and pure glycerol. The engineered strain Y-11 can utilize crude glycerol well and is not affected by the contaminants in crude glycerol, where the highest Y_ERY_ and Q_ERY_ were obtained (Table 1). This result demonstrated that by using crude glycerol as the carbon source, the erythritol titer, Y_ERY_ and Q_ERY_ without the generation of undesirable byproducts were comparable to the reported yields with microorganisms used in commercial erythritol production with glucose as substrate (Table 1). Crude glycerol is mainly a by-product of the biodiesel industry, and its usage in the fermentation of erythritol can not only effectively reduce the cost of erythritol production but also solve the waste disposal issue facing the biodiesel sector.

**Table 1:** Comparison of erythritol titers, yields, and productivities in various *Yarrowia lipolytica* strains.

Microorganism	Strategy	Mode of process	Carbon source	Erythritol (g/L)	Y_ERY_ (g/g)	Q_ERY_ (g/L/h)	References
*Y. lipolytica*	Isolated, purified, and characterized two novel *ER* enzymes of *Y. lipolytica*	Batch bioreactor	Glucose	190	0.63	2.4	Carly et al. (2017)
*Y. lipolytica*	Overexpression of *ZWF1* and *GND1* Disruption of *YlMDH2* and *YlEYD*	Batch bioreactor	Glucose	190	0.63	1.97	Gietz and Schiestl (2007)
*Y. lipolytica*	Ultraviolet mutagenesis and medium optimization	Shake flask batch culture	Glucose	39.24	25.06	-	Wang et al. (2015)
*Y. lipolytica* MK1	Ultraviolet mutagenesis and optimal C: N ratio	Chemostat culture	Pure glycerol	113.1	0.57	1.1	Saran et al. (2015)
*Y. lipolytica* Wratislavia K1	Acetate-negative mutant	Repeated batch culture	Pure glycerol	220	0.43	0.54	Cheng et al. (2018)
*Y. lipolytica* Wratislavia K1	Nitrogen sources optimization	Chemostat culture	Pure glycerol	103	0.52	1.12	Rakicka et al. (2017)
*Y. lipolytica*	Overexpression of codon-optimized bacterial hemoglobin from *Vitreoscilla stercoraria*	Bioreactor culture	Pure glycerol	55	0.37	0.38	Jagtap et al. (2021)
*Y. lipolytica* CICC1675	Osmotic pressure control strategy	One-stage fed-batch fermentation	Pure glycerol	194	0.49	0.95	Yang et al. (2014)
*Y. lipolytica*	Overexpression of four genes, *TKL1*, *TAL1*, *ZWF1*, and *GND1*	Batch bioreactor	Pure glycerol	62.5	0.42	0.62	Mirończuk et al. (2017)
*Y. lipolytica*	Mineral supplementation (manganese ion)	Bioreactor culture	Pure glycerol	47.1	0.47	0.87	Ghezelbash et al. (2014)
*Y. lipolytica* Wratislavia K1	Medium optimization	Fed-batch culture	Pure glycerol	132	0.44	1.01	Tomaszewska et al. (2014)
*Y. lipolytica* Wratislavia K1	Addition of Span 20 surfactant	Fed-batch culture	Pure glycerol	142	0.47	1.1	Rywińska et al. (2015)
*Y. lipolytica*	Overexpression of gene YALI0F18590g encoding the erythrose reductase	Batch culture	Pure glycerol	78.1	0.52	1.0	Rakicka et al. (2016b)
*Y. lipolytica* Wratislavia K1	Acetate-negative mutant	Repeated batch culture	Crude glycerol	155	0.56	0.3	Cheng et al. (2018)
*Y. lipolytica* Wratislavia K1	Nitrogen sources optimization	Chemostat culture	Crude glycerol	81.9	0.40	0.9	Rakicka et al. (2017)
*Y. lipolytica*	Osmotic pressure control strategy Overexpression of native glycerol kinase (*GK*) and transketolase (*TKL*)	Shake flask experiment	Crude glycerol	16.7	-	0.17	Bilal et al. (2020)
*Y. lipolytica* Wratislavia K1	Nitrogen-limited conditions	Fed-batch culture	Crude glycerol	81	0.32	0.48	Rymowicz et al. (2008)
*Y. lipolytica* Wratislavia K1	Glycerol medium with 2.5% NaCl supplementation	Shake flask experiment	Crude glycerol	80	0.49	1.0	Tomaszewska et al. (2012)
*Y. lipolytica* Y-11	Overexpression of three genes, *GUT1*, *GUT2*, and *TKL1* Disruption of *EYD1*	Batch bioreactor	Crude glycerol	150	0.62	1.25	This work

## Conclusion

In this study, a combination of UV mutagenesis and rational metabolic engineering was used to improve erythritol production. The erythritol titer of the final engineered strain in the 5-L bioreactor reached 150 g/l with the highest Y_ERY_ and Q_ERY_ of 0.62 g/g and 1.25 g/l/h using crude glycerol as the carbon source. Unexpectedly, it was discovered that utilizing crude glycerol instead of glucose as a carbon source improved the synthesis of erythritol and successfully suppressed the generation of by-products. Furthermore, it provided a recombinant *Y. lipolytica* strain that efficiently utilizes low-cost crude glycerol to synthesize erythritol and related valuable metabolites, realizing the value-added of crude glycerol.

## Data availability statement

The original contributions presented in the study are included in the article/Supplementary material, further inquiries can be directed to the corresponding author.

## Author contributions

SY designed the experiments, wrote, and revised the manuscript. XP, QW, and QL performed the experiments. XZ and RZ discussed and revised the manuscript. ZR participated in results analysis and interpretation and revised the manuscript. All authors read and approved the final manuscript.

## Funding

This work was supported by the National Key Research and Development Program of China (No. 2021YFC2100900), the National Natural Science Foundation of China (Nos. 32071470 and 32100055), the Natural Science Foundation of Jiangsu Province (No. BK20210464), the National first-class discipline program of Light Industry Technology and Engineering (No. LITE2018-06), the Program of Introducing Talents of Discipline to Universities (No. 111-2-06), and Postgraduate Research & Practice Innovation Program of Jiangsu Provence (KYCX22_2370).

## Conflict of interest

The authors declare that the research was conducted in the absence of any commercial or financial relationships that could be construed as a potential conflict of interest.

## Publisher’s note

All claims expressed in this article are solely those of the authors and do not necessarily represent those of their affiliated organizations, or those of the publisher, the editors and the reviewers. Any product that may be evaluated in this article, or claim that may be made by its manufacturer, is not guaranteed or endorsed by the publisher.
