# Supplementary material for: Enhancing erythritol production from crude glycerol in a wild-type Yarrowia lipolytica by metabolic engineering
Source: Front Microbiol. 2022 Nov 21;13:1054243. doi: 10.3389/fmicb.2022.1054243 (PMC9720325; doi:10.3389/fmicb.2022.1054243)
Supplement: Supplementary file 1 [file Data_Sheet_1.pdf]

Additional File 1

Table S1 Strains used in this study

Strains	Description	Source
E. coli		
JM109	recA1, endA1, gyrA96, thi, hsdR17, supE44, relA1, Δ(lac-proAB)/F [traD36, proab$^+$, lacI^q, lacZΔM15]	This lab
Y. lipolytica		
Y. lipolytica-Y01	UV mutagenesis	This work
Y. lipolytica-Y01 -*△Ku70△LEU2*	*Y. lipolytica △Ku70△LEU2*::*hygB*	This work
Y-02	*Y. lipolytica △Ku70△LEU2*::*hygB,* Integrative plasmid pINA1269-*GUT1*	This work
Y-03	*Y. lipolytica △Ku70△LEU2*::*hygB,* Integrative plasmid pINA1269-*GUT2*	This work
Y-04	*Y. lipolytica △Ku70△LEU2*::*hygB,* Integrative plasmid pINA1269-*GUT1-GUT2*	This work
Y-05	*Y. lipolytica △Ku70△LEU2*::*hygB,* Integrative plasmid pINA1269-*TKL1*	This work
Y-06	*Y. lipolytica △Ku70△LEU2*::*hygB,* Integrative plasmid pINA1269-*TAL1*	This work
Y-07	*Y. lipolytica △Ku70△LEU2*::*hygB,* Integrative plasmid pINA1269-*TKL1-TAL1*	This work
Y-08	*Y. lipolytica △Ku70△LEU2*::*hygB,* Integrative plasmid pINA1269-*GUT1-GUT2-TKL1*	This work
Y-09	*Y. lipolytica △Ku70△LEU2*::*hygB,* Integrative plasmid pINA1269-*GUT1-GUT2-TAL1*	This work
Y-10	*Y. lipolytica △Ku70△LEU2*::*hygB,* Integrative plasmid pINA1269-*GUT1-GUT2-TKL1-TAL1*	This work
Y-11	*Y. lipolytica △Ku70△LEU2*::*hygB,△EYD1*::*BleoR,* Integrative plasmid pINA1269- *GUT1-GUT2-TKL1*	This work

Table S2 Plasmids used in this study

Plasmids	Description	Source
pCAS1yl	Constitutive expression of Cas9 and sgRNA in *Yarrowia lipolytica* cells	Addgene (73226)
pCAS1yl-Δ*Ku70*	*Ku70* Guide RNA module in pCAS1yl	This work
pCAS2yl-Δ*Ku70*	1020bp *hygB* donor DNA in pCAS1yl-Δ*Ku70*	This work
pCAS1yl-Δ*LEU2*	*LEU2* Guide RNA module in pCAS1yl	This work
pCAS2yl-Δ*LEU2*	1020bp *hygB* donor DNA in pCAS1yl-Δ*LEU2*	This work
pCAS2yl-Δ*Ku70*Δ*LEU2*	*LEU2* sgRNA expression cassette cascaded with the *Ku70* sgRNA expression cassette in pCAS2yl-Δ*Ku70*	This work
pCAS1yl-Δ*EYD1*	*EYD1* Guide RNA module in pCAS1yl	This work
pCAS2yl-Δ*EYD1*	375bp *BleoR* donor DNA in pCAS1yl-Δ*EYD1*	This work
pINA1269	*Y. lipolytica* integrative plasmid, hp4d promoter, XPR2 terminator, *leu2* selection marker, AmpR	(Madzak et al. 2000)
pINA1269- *hygB*	pINA1269 plasmid containing *hygB*	This work
pINA1269-*BleoR*	pINA1269 plasmid containing *BleoR*	This work
pINA1269-*GUT1*	pINA1269 plasmid containing *GUT1* from *Y. lipolytica*	This work
pINA1269-*GUT2*	pINA1269 plasmid containing *GUT2* from *Y. lipolytica*	This work
pINA1269-*TKL1*	pINA1269 plasmid containing *TKL1* from *Y. lipolytica*	This work
pINA1269-*TAL1*	pINA1269 plasmid containing *TAL1* from *Y. lipolytica*	This work
pINA1269-*GUT1-GUT2*	pINA1269 plasmid containing *GUT1-GUT2* from *Y. lipolytica*	This work
pINA1269-*TKL1-TAL1*	pINA1269 plasmid containing *TKL1-TAL1* from *Y. lipolytica*	This work
pINA1269-*GUT1-GUT2-TKL1*	pINA1269 plasmid containing *GUT1-GUT2-TKL1* from *Y. lipolytica*	This work
pINA1269-*GUT1-GUT2-TAL1*	pINA1269 plasmid containing *GUT1-GUT2-TAL1* from *Y. lipolytica*	This work
pINA1269-*GUT1-GUT2-TKL1-TAL1*	pINA1269 plasmid containing *GUT1-GUT2-TKL1-TAL1* from *Y. lipolytica*	This work

Table S3 Primers used in this study

Primers	Sequence (5' to 3')
Ku70-sg-1 F	GGGTCGGCGCAGGTTGACGTTGATAGAGTGCTGAAAAGGCGTTTTAGAGCTAGAAATAGC
Ku70-sg-1 R	GCTATTTCTAGCTCTAAAACGCCTTTTCAGCACTCTATCAACGTCAACCTGCGCCGACCC
Ku70-UP F	CATGATTACGCCAAGCTTGTTTCACTACACTACATAACTTGTACCATTCTACCC
Ku70-UP R	TTTCAAAAAGCGGCGGTTCGTG
Ku70-DOWN F	CTAGGGAGGCACATCTAAACGAATAACG
Ku70-DOWN R	AACCCGGTCTCTGTTTAAACAGTGAACGACCAAGACTAAAGGGTG
hygB F	GAACCCGAAACTAAGGATCCATGCCTGAACTCACCGCG
hygB R	CTCGTCCGAGGGCAAAGGAATAGGGTACCTCCATGGCCTGTCC
hp4d- *hygB*-XPR2 F	CACGAACCGCCGCTTTTTGAAAGTAGTAGGTTGAGGCCGTTGAGC
hp4d- *hygB*-XPR2 R	CGTTATTCGTTTAGATGTGCCTCCCTAGACACGGGCATCTCACTTGC
LEU2-sg-1 F	GGGTCGGCGCAGGTTGACGTGGGACATACGAGATCGTCAAGTTTTAGAGCTAGAAATAGC
LEU2-sg-1 R	GCTATTTCTAGCTCTAAAACTTGACGATCTCGTATGTCCCACGTCAACCTGCGCCGACCC
LEU2-UP F	CATGATTACGCCAAGCTTGTTTCAAAGCGAAGAGAAGAGCGGG
LEU2-UP R	GACAGCAACTACTCCTTTCACCAACC
LEU2-DOWN F	GAGACGGTAAGTTGGAGGGGTTTG
LEU2-DOWN R	AACCCGGTCTCTGTTTAAACCGCCAAAGACCAGTGCCAAAC
LEU2 F	AGAGACCGGGTTGGCGG
LEU2 R	CCTTCGGCCCTTTTGGGTTT
EYD1-sg-1 F	GGGTCGGCGCAGGTTGACGTCCAGAACATTGCCGAGACCCGTTTTAGAGCTAGAAATAGC
EYD1-sg-1 R	GCTATTTCTAGCTCTAAAACGGGTCTCGGCAATGTTCTGGACGTCAACCTGCGCCGACCC

EYD1-UP F	AGCAAACATCAACACTCTCCAAGAGC
EYD1-UP R	CAAATGGTTTCTTCAGCCGCTACT
EYD1-DOWN F	GGTAAGCTCCTCATCCCGACTG
EYD1-DOWN R	TGCTGGCCAATTTCACTTACAGAGCACATGA
BleoR F	GCTTATCATCGATGATAAGCATGGCCAAGTTGACCAGTGCCG
BleoR R	GGCCGAGGAGCAGGACTGATTCTTGAAGACGAAAGGGCC
hp4d-*BleoR* -XPR2 F	CACGAACCGCCGCTTTTTGAAAGTAGTAGGTTGAGGCCGTTGAGC
hp4d- *BleoR*-XPR2 R	CGTTATTCGTTTAGATGTGCCTCCCTAGACACGGGCATCTCACTTGC
GUT1 F	CAACCACACACATCCACGTGATGTCTTCCTACGTAGGAGCTCTCG
GUT1 R	GGACAGGCCATGGAGGTACCTTACTCAAGCCAGCCAACAGC
hp4d-*GUT2*-XPR2t-F	CACGAACCGCCGCTTTTTGAAAGTAGTAGGTTGAGGCCGTTGAGC
hp4d-*GUT2*-XPR2t-R	CGTTATTCGTTTAGATGTGCCTCCCTAGACACGGGCATCTCACTTGC
GUT2 F	CAACCACACACATCCACGTGATGTTCAGAACCATTCGAAAACCCG
GUT2 R	GGACAGGCCATGGAGGTACCTTATTTGTCCTTGGGGGTAAGGCCC
hp4d-*TKL1*-XPR2t-F	CACGAACCGCCGCTTTTTGAAAGTAGTAGGTTGAGGCCGTTGAGC
hp4d-*TKL1*-XPR2t-R	CGTTATTCGTTTAGATGTGCCTCCCTAGACACGGGCATCTCACTTGC
TKL1 F	CAACCACACACATCCACGTGATGGCTCCCCAATTTTCAAAGACTG
TKL1 R	GGGGACAGGCCATGGAGGTACCTTAGACACCGTGGCCGGG
hp4d-*TAL1*-XPR2t-F	CACGAACCGCCGCTTTTTGAAAGTAGTAGGTTGAGGCCGTTGAGC
hp4d-*TAL1*-XPR2t-R	CGTTATTCGTTTAGATGTGCCTCCCTAGACACGGGCATCTCACTTGC
TAL1 F	ATACAACCACACACATCCACGTGATGTCTTCCAACTCTCTTGAACAGCT
TAL1 R	GGACAGGCCATGGAGGTACCCTAAGCGGAGAGCTTGGTCTCAAT

Antibiotics selections

Antibiotics are the first choice of gene manipulation markers for wild strains. To identify the available antibiotics, we performed antibiotic tolerance analysis and finally determined that *Y. lipolytica* Y01 was well tolerated to hygromycin B and bleomycin. It can be seen from Figure S1 that hygromycin B 400 μg/ml can inhibit the growth of *Y. lipolytica* Y01, and bleomycin 700 μg/ml can inhibit the growth of *Y. lipolytica* Y01.

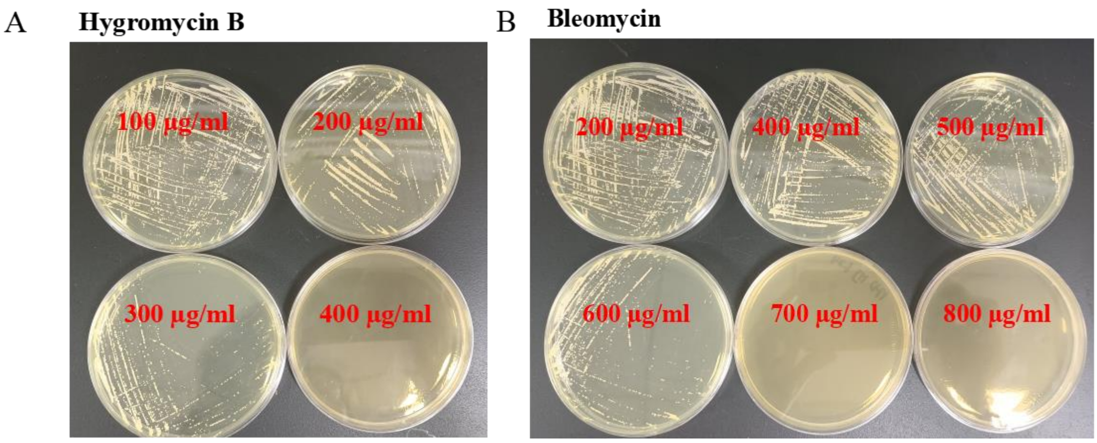

Figure S1: Screening of antibiotic markers of *Y. lipolytica* Y01. A. Screening of hygromycin B concentration for *Y. lipolytica* Y01; B. Screening of bleomycin concentration for *Y. lipolytica* Y01.
